# An Artefactual Cluster of *Mycobacterium abscessus* Pneumonia among Cancer Patients Arising from Contamination

**DOI:** 10.3390/pathogens11010108

**Published:** 2022-01-17

**Authors:** Giusy Diella, Giuseppina Caggiano, Francesco Triggiano, Marco Lopuzzo, Francesca Apollonio, Michele Mastria, Luigi Ronga, Lucia Bonadonna, Luca Lucentini, Antonia Pece, Pietro Milella, Maria Teresa Montagna

**Affiliations:** 1 Department of Biomedical Science and Human Oncology, University of Bari Aldo Moro, Piazza G. Cesare 11, 70124 Bari, Italy; giusy.diella@uniba.it (G.D.); francesco.triggiano@uniba.it (F.T.); marcolopuzzo@gmail.com (M.L.); francesca.apollonio@uniba.it (F.A.); mariateresa.montagna@uniba.it (M.T.M.); 2 Department Interdisciplinary of Medicine, University of Bari Aldo Moro, Piazza G. Cesare 11, 70124 Bari, Italy; mhlmst1992@gmail.com; 3 Microbiology and Virology Unit, AOU Policlinico of Bari, 70124 Bari, Italy; rongalu@yahoo.it; 4 Department of Environment and Health, Istituto Superiore di Sanità, Viale Regina Elena, 00161 Rome, Italy; lucia.bonadonna@iss.it (L.B.); luca.lucentini@iss.it (L.L.); 5 I.R.C.C.S. Istituto Tumori “Giovanni Paolo II” of Bari, Viale Orazio Flacco 65, 70124 Bari, Italy; peceantonella@yahoo.it (A.P.); p.milella@me.com (P.M.)

**Keywords:** *Mycobacterium abscessus* complex, bronchoscope, public health, broncho-lavage

## Abstract

The *Mycobacterium* *abscessus* complex (MABC) is a group of rapidly growing, nontuberculous mycobacteria that are ubiquitous in soil, urban water pipes, swimming pools, and drinking water. Members of the MABC are considered opportunistic pathogens. The aim of this study was to investigate the origins of MABC detected in broncho-lavage (BL) samples from asymptomatic cancer patients. We turned our attention to washing and disinfection procedures for bronchoscopes; we also assessed water and disinfectant samples. Of 10 BL and 34 environmental samples tested, four BL samples (40%) and seven environmental samples (20.6%) tested positive for MABC. We hypothesized that contamination could arise from the prewashing machine and/or the water used because no patient had clinical or radiological signs consistent with MABC respiratory tract infection. Our study highlights the importance of evaluating cleaning and disinfection procedures for endoscope channels to reduce the potential spread of microorganisms and artefactual results arising from contamination.

## 1. Introduction

The *Mycobacterium abscessus* complex (MABC) is a group of rapidly growing, nontuberculous mycobacteria (NTM) that includes three subspecies: *M*. *abscessus,* M. *massiliense*, and *M*. *bolletii.* Members of the MABC are ubiquitous in soil, urban water pipes (sometimes in symbiosis with amoebae) [1], swimming pools [2,3], and drinking water, where they can remain viable after treatment and disinfection [4].

Although not highly virulent, similarly to other NTM such as *M. chimaera* belonging to the *Mycobacterium avium* complex [5], members of the MABC are considered opportunistic pathogens and can cause disseminated infections in immunocompromised hosts. Examples include bacteraemia following dialysis catheter use [6], central nervous system infections, and skin and soft tissue diseases [7]. Skin and soft tissue infections generally arise from disseminated disease or from direct contact with contaminated objects or fluids through traumatic injuries or surgical wounds. Infections by MABC members have been reported in patients undergoing cosmetic procedures (e.g., mesotherapy), tattooing, and acupuncture [8].

Members of the MABC are responsible for up to 80% of NTM respiratory infections worldwide. Thus, members of the MABC are becoming prominent and worrisome pathogens and are frequently in patients with human immunodeficiency virus infection, chronic obstructive pulmonary disease, bronchiectasis, and cystic fibrosis [4,7,9,10]. In these patients, MABC mycobacteriosis can be very serious and often has fatal outcomes. Outbreaks can pose serious risks in centres specializing in lung transplantation and treatment of cystic fibrosis [11]. Moreover, treatment of MABC infections is complicated because these bacteria are notoriously resistant to standard antituberculosis agents [12]. Infections are often relentless despite treatment, and even when treatment is apparently effective, relapse is common [13]. This infection can be acquired in community or hospital settings. In community settings, transmission is mainly associated with contaminated water networks, while in hospital settings transmission can be also related to contaminated disinfectants, saline, and electromedical devices [11,12,13,14,15].

The epidemiology of MABC infection is complex and varies by geographic area and patient type. Among rapidly growing mycobacteria, the MABC is recognized as the most pathogenic. Multicentre studies have reported prevalence ranging from 2.7% in Europe to 14% in the United States, where MABC infections are the second most common mycobacterial infections after *M. avium* infections [12,16,17,18]. In the Russian Federation, MABC species were isolated from 3.1% of examined patients and the prevalence among patients with cystic fibrosis was 1.5% [19]. In Taiwan, members of the MABC comprise 17.2% of all clinical NTM isolates, equivalent to 1.7 cases per 100,000 population [20].

The aim of this study was to investigate the origin of a presumed cluster of mycobacteriosis resulting from MABC detected in broncho-lavage (BL) samples from asymptomatic patients with cancer.

## 2. Results

Overall, of 44 samples tested (10 BL and 34 environmental), 11 (25%) were positive for MABC: four BL samples (4/10, 40%) and seven environmental samples (7/34, 20.6%) (Table 1). Positive samples included:

Two water samples from tap water after purification filtering;

One sample of prewashing solution with disinfectant that had not been passed through a sterile bronchoscope;

Two prewashing solution samples after passing through a sterile bronchoscope;

Two samples of prewashing solution with disinfectant after passing through a sterile bronchoscope.

For Patient No. 1, a control bronchoscopy with a disposable bronchoscope was negative for MABC.

## 3. Discussion

Microbiological surveillance conducted among patients at high risk of infection often highlights infectious complications of bacterial and fungal origin that are sometimes difficult to resolve [21,22]. Diseases caused by NTM are generally uncommon. However, NTM can cause infections in immunocompromised individuals as they are ubiquitous microorganisms in the environment and in engineered environmental habitats [1,4,23].

Because the control bronchoscopy of Patient No. 1 resulted negative for MABC, the suspicion that there was environmental contamination rather than an infection or colonization of the patient was corroborated. Therefore, due to the oncological pathologies of the patients often also being platelet and neutropenic, to perform follow-up for other patients was not considered appropriate, as bronchoscopy is an invasive diagnostic test.

According to the manufacturing protocol in our study, the instruments dedicated to sanitization of bronchoscopes are washed and sanitized in the prewash machine, then sterilized using strong disinfectants (acetic acid and hydrogen peroxide). Although all these steps were carried out, MABC species were isolated from the water samples used in the prewash phase of the bronchoscope.

In this paper, we discussed a “presumed” cluster of mycobacteriosis as we detected more positive samples in a short period. We hypothesized that contamination arose from the prewashing machine and/or from the water used because no patients had clinical or radiological signs consistent with MABC respiratory tract infection. Probably, the bronchoscope was contaminated during the prewashing phase but did not transfer mycobacteria to patients because the part of the bronchoscope in direct contact with the mucosa was sterile and disposable.

Bronchoscopes are difficult to clean and disinfect. These instruments are fragile, have a complex design [24], and cannot be subjected to thermal sterilization processes because of the presence of cameras, optical fibres, and probes. Therefore, their sanitization is carried out exclusively using strong disinfectants that may be insufficient to eliminate MABC species.

NTM are difficult to eradicate via common decontamination practices and are highly resistant to standard disinfectants such as chlorine [3]. Among disinfectants, acetic acid appears to have significant mycobactericidal activity [25] and hydrogen peroxide is active against a wide range of microorganisms including methicillin-resistant *Staphylococcus aureus*, vancomycin-resistant *Enterococcus*, *Mycobacterium tuberculosis*, spores, viruses, and multidrug-resistant Gram-negative bacilli [26,27,28]. However, other studies have demonstrated the difficulty of eliminating clinical MABC isolates, which can persist after exposure to several biocides commonly used in hospitals [29]. *M. abscessus sensu stricto* is effectively killed by exposure to 6% acetic acid for 30 min, while *M. bolletii* and *M. massiliense* appear more resistant [25].

In our study, acetic acid-based and hydrogen peroxide-based compounds were used for the disinfection/sterilization procedure. However, the effectiveness of disinfection, as well as the efficacy of antibiotic therapies in patients with lung diseases, may differ for members of the MABC, especially if these bacteria are present in biofilms. This matrix plays an essential role in MABC infections: the formation of biofilm-like microcolonies was detected in the lungs of patients with mycobacteriosis [24,30].

Members of the MABC exhibit two distinct morphotypes: a rough one, devoid of surface-associated glycopeptidolipids, and a smooth one. These two types differ in their virulence and pathogenicity, as well as in their ability to form biofilms [31]. Smooth colony variants colonize the pulmonary airways in a biofilm, and after spontaneously losing surface glycopeptidolipids, morphologically change into the rough colony type, causing inflammation and invasive disease [30]. Rough colonies of MABC species form biofilms with a higher degree of mechanical strength than biofilms produced from smooth type colonies [30].

Water is known to be an important environmental reservoir of mycobacteria. In water pipes and engineered environmental habitats, biofilm formation represents a successful survival strategy for mycobacteria [3]. Some authors have identified MABC species both in hospital water and in endoscopic cleaning equipment [13], but could not resolve doubts regarding the true source of these bacteria. In many cases, it is not clear whether the source of outbreaks is an infected patient or water. Similarly, based on our analyses, we cannot say with certainty that the water used in the prewashing phase was the source of MABC. The samples taken directly from the tap connected to the water supply were negative for MABC.

We isolated MABC species from water samples derived from a filtration system installed to prevent bacteria spread in water systems, especially *Legionella* species. Levels of MABC contamination in the water supply were probably low, but the filter may have concentrated the bacteria to detectable quantities. For this reason, the hospital removed and replaced the filter to ensure patient safety. The manufacturer has withdrawn the filter for specific checks and analysis.

Although MABC members are generally ubiquitous and their isolation from the respiratory tract does not always indicate a state of disease [32], the potential for person-to-person transmission makes identification at the subspecies level mandatory because of differences in antibiotic susceptibility profiles and treatment outcomes. Additionally, MABC infections are difficult to treat because these organisms are resistant to conventional tuberculosis therapies and most antimicrobial agents [33,34].

Future studies will assess the sensitivity of MABC members to disinfectants. Furthermore, the role of antibacterial water system filters in the management of waterborne diseases is an area for further study. Such filters may eliminate the spread of *Legionella*, but less effectively reduce the spread of other bacteria [35].

## 4. Materials and Methods

### 4.1. Study Design

One of the hospitals in the Apulia region (Southern Italy) is devoted exclusively to the care of patients with cancer, in its various forms and stages. To assess their clinical status, this hospital, equipped with 109 beds, follows a protocol that provides, among other investigations, microbiological surveillance. In this context, during scheduled daytime hospital check-up, patients are tested for the presence of fungi, viruses, and bacteria (including *Mycobacterium* species). The materials used for testing are samples obtained using bronchoscopes. After use, the bronchoscopes undergo sanitation protocols including decontamination in a prewash machine and sterilization with strong disinfectants in a sterilizer. Bronchoscopy instruments are sanitized in accordance with the ISO 15883–4 [36] and ISO 14937 [37] standards using a concentrated and water-soluble detergent (containing protease and isopropyl alcohol) and disinfectant solutions based on acetic acid (1%) and hydrogen peroxide (1%).

### 4.2. Detection of Mycobacteria

From 1 December 2020 to 5 January 2021, 10 BL samples were examined from the same number of patients. These patients were not hospitalized and had no symptoms of pneumonia.

Prior to testing for mycobacteria, BL samples were pre-treated and homogenized with *N*-acetyl-l-cysteine/4% sodium hydroxide/1.47% sodium citrate. Subsequently, samples were inoculated in both liquid and solid media. *Mycobacterium* growth-indicator tubes (BD MGIT-960, BD, Sparks, MD, USA) containing oleic acid/albumin/dextrose/catalase and polymyxin B/azlocillin/nalidixic acid/antibiotics including trimethoprim (BD; 5.0 mcg/mL) were used for culture. The decontaminated samples (0.5 mL) were inoculated into the culture media. The culture tubes were incubated for 42 days at 37 °C. A detector read the fluorescence that a sensor placed on the bottom of the tubes emitted if stimulated by the reduction of oxygen arising from bacterial proliferation.

Tubes from Löwenstein–Jensen (BD) were used as a solid medium. Tubes were inoculated with 0.5 mL of decontaminated samples, incubated for 70 days at 37 °C, and evaluated weekly. The presence of suspected acid-alcohol-fast bacilli was evaluated by microscopic observation after Ziehl–Neelsen staining.

Finally, an immunochromatographic test (BD MGIT TBc identification test) was used to evaluate the presence of *Mycobacterium tuberculosis*. The GenoType Mycobacterium CM v2.0 kit (HAIN Lifescience GmbH, Nehren, Germany) was used according to the manufacturer’s instructions to identify NTM.

### 4.3. Environmental Sampling

To identify the source of NTM, we turned our attention to bronchoscopes and procedures for their washing and disinfection. To this end, we examined water and disinfectant samples. Investigations were performed in a room dedicated to procedures for sanitation and sterilization of bronchoscopy instruments. To assess the effectiveness of all the steps in the reclamation procedures, the decontamination and disinfection processes were simulated using a bronchoscope that has been disinfected and sterilized according to the protocol in the hospital.

In total, 34 samples were collected: six drinking water samples (including four from taps without filtering systems used for prewashing and two from taps with filtering systems to purify water before entering the sterilizer), nine prewashing solutions, five sterilization solutions, nine samples of detergents and disinfectants from different production lots, and five samples of sterile physiologic solution passed into sterile bronchoscopes.

For the water supply investigation, cold water was allowed to flow for 3 min before sampling [38] after disinfecting the taps with 10% sodium hypochlorite solution followed by flaming. Water (1 L) was collected in sterile containers containing sodium thiosulfate pentahydrate (0.01% w/v), transported at a controlled temperature (4 °C), and analysed within 48 h. Each sample was filtered through a cellulose membrane (Ø 47 mm) with a pore size of 0.45 µm (Millipore, Milan, Italy). This membrane was suspended in 10 mL of the same water, vortexed, and assessed for the presence of MABC. Each concentrated sample (5 mL) was treated with N-acetyl-L-cysteine/4% sodium hydroxide/1.47% sodium citrate (1:1) to eliminate environmental bacteria. Subsequently, the sample was centrifuged at 13,000× *g* for 15 min. The supernatants were removed and 0.5 mL of the remaining suspensions were inoculated on both liquid and solid media and incubated as described above. The presence of MABC was evaluated by microscopic observation of acid-alcohol-fast bacilli after Ziehl–Neelsen staining followed by hybridization using the GenoType Mycobacterium CM v2.0 kit (HAIN Lifescience GmbH).

Disinfectants and detergents employed in bronchoscope remediation processes were also investigated. Each sample (100 mL) was centrifuged at 13,000× *g* for 15 min and the supernatant was removed. Because of the small pellets obtained, the samples were inoculated in both liquid and solid medium without previous decontamination and processed in the same manner as the water samples.

## 5. Conclusions

Our study highlights the importance of evaluating cleaning and disinfection procedures for endoscope channels. To reduce the potential spread of microorganisms, hospital infection committees should implement timely interventions for tracing sources of infection and should constantly update infection control and prevention measures. Assessing the presence of biofilms within endoscopes should be a priority in disinfection procedures.

Monitoring of all medical devices that could be potential sources of infection, including gastrointestinal endoscopes, eye contact instruments, and heater–cooler units for cardiac surgery, should be included in hospital infection prevention programs. Such interventions would reduce the need for medical treatment and its economic impact.

## Figures and Tables

**Table 1:** Environmental samples tested for *Mycobacterium abscessus* complex (MABC).

Sample (N)	Samples Positive for MABC *n* (%)
Phase 1: prewashing process	
Water from tap without a filtration system (4)	0
Prewashing solution (1)	0
Prewashing solution with a disinfectant (2)	1 (50)
Prewashing solution using a sterile bronchoscope (2)	2 (100)
Prewashing solution with a disinfectant using a sterile bronchoscope (2)	2 (100)
Rewashing water (2)	0
Phase 2: sterilization process	
Water from tap with a filtration system (2)	2 (100)
Sterilization solutions (5)	0
Detergents and disinfectants (9)	0
Sterile physiologic solution passed through a sterile bronchoscope (5)	0
Total (34)	7 (20.6)

Abbreviations: MABC = *Mycobacterium abscessus* complex.

## Data Availability

Data are contained within the article.

## References

[1] King D.N., Donohue M.J., Vesper S.J., Villegas E.N., Ware M.W., Vogel M.E., Furlong E.F., Kolpin D.W., Glassmeyer S.T., Pfaller S. (2016). Microbial pathogens in source and treated waters from drinking water treatment plants in the United States and implications for human health. Sci. Total Environ..

[2] D’Ancona F.P., Kanitz E.E., Marinelli L., Sinagra J.L., Prignano G., Cerocchi C., Bonadonna L., Tortoli E., Capitanio B., Cottarelli A. (2014). Non tuberculous cutaneous mycobacteriosis in a primary school in Rome: Epidemiological and microbiological investigation. Ann. Ig..

[3] Briancesco R., Meloni P., Semproni M., Bonadonna L. (2014). Non-tuberculous mycobacteria, amoebae and bacterial indicators in swimming pool and spa. Microchem. J..

[4] Degiacomi G., Sammartino J.C., Chiarelli L.R., Riabova O., Makarov V., Pasca M.R. (2019). *Mycobacterium abscessus*, an emerging and worrisome pathogen among cystic fibrosis patients. Int. J. Mol. Sci..

[5] Riccardi N., Monticelli J., Antonello R.M., Luzzati R., Gabrielli M., Ferrarese M., Codecasa L., Di Bella S., Giacobbe D.R. (2020). Mycobacterium chimaera infections: An update. J. Infect. Chemother..

[6] Jheeta A.S., Rangaiah J., Clark J., Makanjuola D., Somalanka S. (2020). *Mycobacterium abscessus*—An uncommon, but important cause of peritoneal dialysis-associated peritonitis—Case report and literature review. BMC Nephrol..

[7] Martiniano S.L., Nick J.A., Daley C.L. (2019). Nontuberculous mycobacterial infections in cystic fibrosis. Thorac. Surg. Clin..

[8] Kothavade R.J., Dhurat R.S., Mishra S.N., Kothavade U.R. (2013). Clinical and laboratory aspects of the diagnosis and management of cutaneous and subcutaneous infections caused by rapidly growing mycobacteria. Eur. J. Clin. Microbiol. Infect. Dis..

[9] Andrew E.C., Connell T., Robinson P., Curtis N., Massie J., Robertson C., Harrison J., Shanthikumar S., Bryant P.A., Starr M. (2019). Pulmonary *Mycobacterium abscessus* complex in children with cystic fibrosis: A practical management guideline. J. Paediatr. Child Health.

[10] Mougari F., Guglielmetti L., Raskine L., Sermet-Gaudelus I., Veziris N., Cambau E. (2016). Infections caused by *Mycobacterium abscessus*: Epidemiology, diagnostic tools and treatment. Expert Rev. Anti-Infect. Ther..

[11] Bryant J.M., Grogono D.M., Greaves D., Foweraker J., Roddick I., Inns T., Reacher M., Haworth C.S., Curran M.D., Harris S.R. (2013). Whole-genome sequencing to identify transmission of *Mycobacterium abscessus* between patients with cystic fibrosis: A retrospective cohort study. Lancet.

[12] Lee M.R., Sheng W.H., Hung C.C., Yu C.J., Lee L.N., Hsueh P.R. (2015). *Mycobacterium abscessus* complex infections in humans. Emerg. Infect. Dis..

[13] Thomson R., Tolson C., Sidjabat H., Huygens F., Hargreaves M. (2013). *Mycobacterium abscessus* isolated from municipal water—A potential source of human infection. BMC Infect. Dis..

[14] Viana-Niero C., Lima K.V., Lopes M.L., Rabello M.C., Marsola L.R., Brilhante V.C., Durham A.M., Leão S.C. (2008). Molecular characterization of *Mycobacterium massiliense* and *Mycobacterium bolletii* in isolates collected from outbreaks of infections after laparoscopic surgeries and cosmetic procedures. J. Clin. Microbiol..

[15] Benwill J.L., Wallace R.J. (2014). *Mycobacterium abscessus*: Challenges in diagnosis and treatment. Curr. Opin. Infect. Dis..

[16] Martiniano S.L., Sontag M.K., Daley C.L., Nick J.A., Sagel S.D. (2014). Clinical significance of a first positive nontuberculous mycobacteria culture in cystic fibrosis. Ann. Am. Thorac. Soc..

[17] Adjemian J., Olivier K.N., Prevots D.R. (2014). Nontuberculous mycobacteria among patients with cystic fibrosis in the United States: Screening practices and environmental risk. Am. J. Respir. Crit. Care Med..

[18] Tissot A., Thomas M.F., Corris P.A., Brodlie M. (2018). Nontuberculous mycobacteria infection and lung transplantation in cystic fibrosis: A worldwide survey of clinical practice. BMC Pulm. Med..

[19] Kalimulina K.R., Ismatullin D.D., Lyamin A.V., Kondratenko O.V., Kozlov A.V., Zhestkov A.V. (2020). *Mycobacterium abscessus* complex representatives in patients with bronchopulmonary pathology: Prevalence, peculiarities of cultivation and identification. Klin. Lab. Diagn..

[20] Lai C.C., Tan C.K., Chou C.H., Hsu H.L., Liao C.H., Huang Y.T., Yang P.C., Luh K.T., Hsueh P.R. (2010). Increasing incidence of nontuberculous mycobacteria, Taiwan, 2000–2008. Emerg. Infect. Dis..

[21] Montagna M.T., Lovero G., De Giglio O., Iatta R., Caggiano G., Montagna O., Laforgia N. (2010). Invasive fungal infections in neonatal intensive care units of Southern Italy: A multicenter regional active surveillance (AURORA Project). J. Prev. Med. Hyg..

[22] Agodi A., Auxilia F., Barchitta M., Brusaferro S., D’Alessandro D., Grillo O.C., Montagna M.T., Pasquarella C., Righi E., Tardivo S. (2013). Trends, risk factors and outcomes of healthcare-associated infections within the Italian network SPIN-UTI. J. Hosp. Infect..

[23] Briancesco R., Semproni M., Paradiso R., Bonadonna L. (2014). Nontuberculous mycobacteria: An emerging risk in engineered environmental habitats. Ann. Microbiol..

[24] Neves M.S., da Silva M.G., Ventura G.M., Côrtes P.B., Duarte R.S., de Souza H.S. (2016). Effectiveness of current disinfection procedures against biofilm on contaminated GI endoscopes. Gastrointest. Endosc..

[25] Cortesia C., Vilchèze C., Bernut A., Contreras W., Gómez K., de Waard J., Jacobs W.R., Kremer L., Takiff H. (2014). Acetic acid, the active component of vinegar, is an effective tuberculocidal disinfectant. mBio.

[26] De Giglio O., Coretti C., Lovero G., Barbuti G., Caggiano G. (2014). Pilot study on the antibacterial activity of hydrogen peroxide and silver ions in the hospital environment. Ann. Ig..

[27] Boyce M.J., Guercia K.A., Sullivan L., Havill N.L., Fekieta R., Kozakiewicz J., Goffman D. (2017). Prospective cluster controlled crossover trial to compare the impact of an improved hydrogen peroxide disinfectant and a quaternary ammonium-based disinfectant on surface contamination and health care outcomes. Am. J. Infect. Control.

[28] Montagna M.T., Triggiano F., Barbuti G., Bartolomeo N., De Giglio O., Diella G., Lopuzzo M., Rutigliano S., Serio G., Caggiano G. (2019). Study on the in vitro activity of five disinfectants against nosocomial bacteria. Int. J. Environ. Res. Public Health.

[29] Caskey S., Moore J.E., Rendall J.C. (2018). In vitro activity of seven hospital biocides against *Mycobacterium abscessus*: Implications for patients with cystic fibrosis. Int. J. Mycobacteriol..

[30] Dokic A., Peterson E., Arrieta-Ortiz M.L., Pan M., Di Maio A., Baliga N., Bhatt A. (2021). *Mycobacterium abscessus* biofilms produce an extracellular matrix and have a distinct mycolic acid profile. Cell Surf..

[31] Kolpen M., Jensen P.Ø., Qvist T., Kragh K.N., Ravnholt C., Fritz B.G., Johansen U.R., Bjarnsholt T., Høiby N. (2020). Biofilms of *Mycobacterium abscessus* complex can be sensitized to antibiotics by disaggregation and oxygenation. Antimicrob. Agents Chemother..

[32] Teri A., Sottotetti S., Arghittu M., Girelli D., Biffi A., D’Accico M., Daccò V., Gambazza S., Pizzamiglio G., Trovato A. (2020). Molecular characterization of *Mycobacterium abscessus* subspecies isolated from patients attending an Italian cystic fibrosis centre. New Microbiol..

[33] Bartolomeo K., Hassanein M., Vachharajani T.J. (2021). Management of peritoneal dialysis *Mycobacterium abscessus* exit-site infection: A case report and literature review. J. Vasc. Access.

[34] Victoria L., Gupta A., Gómez J.L., Robledo J. (2021). *Mycobacterium abscessus* complex: A review of recent developments in an emerging pathogen. Front. Cell. Infect. Microbiol..

[35] Parkinson J., Baron J.L., Hall B., Bos H., Racine P., Wagener M.M., Stout J.E. (2020). Point-of-use filters for prevention of health care-acquired Legionnaires’ disease: Field evaluation of a new filter product and literature review. Am. J. Infect. Control..

[36] ISO (2018). ISO 15883-4:2018, Washer-Disinfectors—Part 4: Requirements and Tests for Washer-Disinfectors Employing Chemical Disinfection for Thermolabile Endoscopes.

[37] ISO (2009). ISO 14937:2009, Sterilization of Health Care Products—General Requirements for Characterization of a Sterilizing Agent and the Development, Validation and Routine Control of a Sterilization Process for Medical Devices.

[38] ISO (2006). ISO 19458:2006, Water Quality—Sampling for Microbiological Analysis.

